# ‘Ref! Could You Help Me?’—Building a Positive Climate by Referees during Floorball Competitions in Youth Sport

**DOI:** 10.3390/ijerph19020979

**Published:** 2022-01-16

**Authors:** Wiesław Firek, Katarzyna Płoszaj, Paweł Gąsior, Ewa Malchrowicz-Mośko

**Affiliations:** 1 Faculty of Physical Education, Józef Piłsudski University of Physical Education in Warsaw, 00-968 Warsaw, Poland; katarzyna.ploszaj@awf.edu.pl; 2 Department of Physical Education and Sport, Podhale State College of Applied Sciences in Nowy Targ, ul. Kokoszków 71, 34-400 Nowy Targ, Poland; pawel.gasior@ppuz.edu.pl; 3 Faculty of Physical Culture Sciences, Eugeniusz Piasecki University of Physical Education in Poznań, 61-871 Poznań, Poland; malchrowicz@awf.poznan.pl

**Keywords:** educational practice, floorball, youth sport, referee, positive climate, situation monitoring

## Abstract

In creating a positive climate in sport for children and youth, the role of adults is of key importance as their behavior and attitudes determine the experiences and multilateral development of young players. Relatively recently, the importance of the referee in creating a supportive sporting environment has begun to be emphasized. This concerns, in particular, team sports in which the referees interact with players many times and influence the course of the game. The aim of the study was to evaluate the quality of the referee–players’ interactions during youth floorball matches in terms of building a positive climate and responsiveness to the players’ needs. Another aim of the study was to examine whether the referee’s qualifications and players’ gender affect the quality of their interactions with the players. The study was conducted among 21 referees officiating matches for girls and boys aged 12–18. Naturalistic and structured observation methods were used in the study. The observation was conducted using a wireless intercom that allows listening to verbal messages directed to the players. Furthermore, the referee’s work was recorded using a camera. The results of the statistical tests did not show any significant differences in the assessment of referees between the groups distinguished in terms of the referees’ license and players’ gender in both examined dimensions. The observations showed that the average rating of building a positive climate by referees during a sporting event measured on a seven-point scale was ‘poor’ (2.81 pts). The referees were assessed significantly higher on the second dimension (responsiveness to the players’ needs), although an overall rating of 3.81 pts means a medium level of interaction quality. The results indicate areas in which referees can improve. They lead to the following conclusions: (i) the contents of training for floorball referees should include problems of pedagogy and developmental psychology; (ii) referees should be equipped with appropriate competencies for building a positive climate during matches and monitoring the players’ needs; (iii) referees appointed to officiate children and youth games should be characterized by appropriate predispositions.

## 1. Introduction

In creating a positive climate in children and youth sports, the role of adults is of key importance as their behavior and attitudes determine the experiences and multilateral development of young players [1]. Previous research on building a positive climate in youth sports has focused on coaches [2,3,4,5,6] and parents [7,8,9]. It was found that for young players to engage in sport (and stay in this environment for a longer period of time), the emotional support of the coach and parents is important as it allows them to express their autonomy, enjoy the game, and have contact with peers [10,11,12]. Relatively recently, the importance of the referee in creating a safe educational environment has begun to be emphasized [13,14,15]. This especially concerns sports such as football, rugby, handball, and floorball, where referees interact with players in numerous ways and can influence the course of the game [16]. Researchers dealing with these problems claim that the referee is an under-researched subject in youth sport [13,14], yet the referee’s behavior and attitudes have a critical effect on the atmosphere and spirit of the game [13,17]. Hence the young player’s question to the referee in the title of the present paper: “Ref! Could you help me?” This question can be extended to ask whether the referee’s role is to emotionally and organizationally support children and youth during sports games. If so, then, in addition to the supervisory and control functions, the referee should also perform a pedagogical function. It is assumed that the quality of the referee–players’ interactions in the dimension of building a positive climate and responding to the players’ needs is one of the basic mechanisms of the referee’s educational interactions [18]. The ability to apply specific methods, techniques, and strategies conducive to building a positive climate during sports competitions is becoming not just a postulate, but an obligation. It results from social expectations [19] and contemporary formulated objectives of youth sport [2,3,10,13,20]. This is particularly important in the face of children and adolescents leaving sports, resulting from lack of enjoyment of the game, poor relationships with peers and coaches, or excessive expectations of achievements [7,21,22,23]. The referee, as the only adult present with the floorball players on the field, should consciously and intentionally engage in educational activities. The educational function of a referee can be defined as follows: “it means an intentional transfer of knowledge and skills as well as ideals and patterns of conduct characteristic of sports culture, expressed primarily in the quality of interactions with the players, aiming to build a positive climate of the sporting event and organize it in such a way that it is a source of positive cognitive, emotional, social, and health experiences for the child. The referee performs this function as part of common educational interventions, whereas the other persons involved are coaches and parents” [13].

A climate conducive to educational practice in youth sport is defined similarly yet termed differently in the literature as a “motivational climate” [10,24,25,26], which is conducive to the completion of specific tasks [27]; “caring climate” [28,29] providing a sense of security and being appreciated and respected [28], being the basis for prosocial behavior [30]; “emotionally safe environment” enhancing the joy and enthusiasm of competition [31], or more generally as “positive climate” determining the positive sporting experience of the athlete [13,32]. Most of these studies have focused on the climate conducive to the child’s undertaking certain activities (tasks) and their outcomes (goals achieved) and the impact of relationship quality on the child’s motivation, behavior, and attitudes towards the coach and peers [10,28,33]. The quality of the interactions between players, coaches, and referees contributing to the intended educational outcomes has been verified to a lesser extent.

The present study supports the presumption that the game climate determines the positive sporting experience of children and young people and therefore promotes better cognitive, emotional, moral, health, and social development [13,29,30]. Researchers are looking for ways to make the sports environment more friendly to young athletes [3,5,20,32,34,35,36]. We posit that a referee is an important link in sports education and he or she can influence the atmosphere of the game by creating a conducive playing environment for the child. Therefore, the research presented here concerns the referee’s interactions with players. These interactions determine the climate of the match to a significant extent [13].

The aim of the study was to evaluate the quality of the referee–players’ interactions during youth floorball games in terms of building a positive climate and responsiveness to the players’ needs during the game. The referees were assessed with reference to the referee-educator model [13], so the aim was to identify areas of the referees’ work that could be improved or enhanced to make a sports match a more effective educational event. It was assumed that a positive climate means the emotional bond between the referee and the players, expressing mutual interest, enthusiastic attitudes, and enjoyment of the interactions, whereas responsiveness means the referee’s reactions to the emotional, cognitive, social, and health needs of the players [18]. For methodological reasons, we also checked whether the referee’s qualifications and players ’gender affect the quality of his or her interactions with the players. The lack of significant statistical differences in this respect would allow the presentation of the results for referees in general. The research was exploratory in nature, which means that the postulated referee’s tasks (performed within the dimensions studied here and considered part of the educational function) are a relatively new subject of research that has been little explored so far [37]. Quantitative research is particularly needed. The disadvantage of exploratory research is that it does not always provide satisfactory answers to research questions and that the answers are inconclusive. So far, sports referees have been studied in terms of performing their educational function in soccer [38], handball [18], and rugby [39]. Our research will allow for the characterization of referees’ activities aimed at using the educational potential of another sport, i.e., floorball. The results of the study can be used to modify the content of referee training by individual sports associations, especially the sports in which the referee is an interactor [16]. High-quality interactions between referees and players can (i) increase the satisfaction of young athletes participating in sports and prevent them from giving up sporting activity; (ii) increase the job satisfaction of referees and respect among children and young people, fans/parents, and coaches; (iii) raise the awareness of adults entering the role of a referee (e.g., former players, coaches) about building positive sporting experiences for young players; and (iv) help organizations working in the field of children and youth sport to develop programs to support building a positive climate in the sporting environment.

## 2. Materials and Methods

### 2.1. Participants

In the 2020/2021 season, there were 47 floorball referees in Poland with a referee license. Of the population of referees, those with a “candidate referee” license were excluded from the study. There were 25 referees officiating youth matches in the age categories studied in the 2020/2021 season. Thus, with a confidence level of alpha = 0.05 and a margin of error of 5%, the sample size was calculated to be 19 referees (sample size calculator, Raosoft, Inc., Seattle, WA, US, 2004). In total, 21 referees were examined. Prior to the study, cooperation was established with the Polish Floorball Federation and its Referee Department. Immediately prior to observation, each referee was informed about the purpose of and procedures used in the study. Referees gave voluntary informed consent to participate in the study by signing a consent form. They were also informed that they could withdraw their consent at any time. The observations were conducted in May and June 2021 among referees officiating matches for girls and boys aged 12–18 in Poland. There were 20 male and 1 female referee. This proportion reflects the ratio of men and women in the population of floorball referees in Poland. The mean age of the subjects was 24.44 years (SD = 4.082; min = 19 years; max = 32 years), while the mean refereeing experience was 6 years (SD = 4.761; min = 1; max = 17). Furthermore, 38% (n = 8) referees had a R-B license (persons who completed a refereeing course of the Polish Floorball Federation and practical experience of performing the role of a referee, confirmed by the observer’s opinion) [40]. The remaining referees (62%, *n* = 13) held a R-A license (individuals with refereeing experience who had officiated matches for at least several seasons across all age categories) [40].

### 2.2. Measure

Naturalistic and structured observation methods were used in the study. For the naturalistic observation method, the researcher makes observations of the subjects’ behavior in natural situations. In this case, this occurs while refereeing youth matches. Structured observation is similar to the naturalistic observation in that naturally occurring behavior is observed. However, the emphasis here is on collecting quantitative rather than qualitative data. The researcher is interested in a limited group of behaviors that are closely described on the observation sheet rather than in all behaviors. This allows for the quantification of the behaviors that are observed [37,41,42]. This study used a direct observation tool to assess the quality of referee–players’ interactions in children and youth sport R-PIASS (referee–players’ interaction assessment scoring system) [18]. Due to the aims of the study, the referee’s interactions with the players in terms of building a positive climate and responding to the players’ needs were observed. Both dimensions can be called the emotional support given to the players by the referee. A detailed description of the evaluated indicators in terms of the referee building a positive climate is presented in Table 1, whereas the description in terms of responding to the player’s needs is shown in Table 2.

### 2.3. Procedure

Observations were conducted using a wireless intercom (EJEAS Fbim, EJEAS Technology Co., Ltd., Shenzhen, China) that allows listening to verbal messages directed to the players. Furthermore, the referee’s work was recorded using a GoPro HERO8 Black camera (GoPro, Inc., San Mateo, CA, USA). All observations were made by a single observer. The referee’s behavior immediately before the match, during the match, and immediately after the match was observed. The observer observed the referee–players’ interactions as determined by the R-PIASS tool in the dimensions of building a positive climate and responsiveness to players’ needs by taking notes. After each observation, the observer replayed the match again from the video recording to check whether he had not missed any significant behavior of the referee. Then, the observer coded the results in the observation sheet. Each indicator from the dimensions shown in Table 1 and Table 2 was scored on a three-point poor-average-good scale. Based on the scores for each indicator, the scores for the positive climate and responsiveness dimensions were determined according to the instructions contained in Table 3.

### 2.4. Analytic Strategy

Data were analyzed using basic descriptive statistics such as percentages, means, medians, skewness, and kurtosis. The normality of the distribution of variables was verified using the Shapiro–Wilk test. The significance of the differences between indicators was tested using the Chi-squared test. The non-parametric Mann–Whitney U test for two independent samples was used to assess differences in ratings of the quality of referee–players’ interactions in groups distinguished by the referee license held and children’s gender. Furthermore, Wilcoxon’s signed-rank test was employed to assess the quality of the interactions between the two dependent variables studied (building a positive climate and responsiveness). The sample size was determined using the sample size calculator (Raosoft, Inc., Seattle, WA, US, 2004). The level of statistical significance was set at 0.05. The reliability of the R-PIASS tool was tested by Cronbach’s alpha coefficient. Calculations were performed using the PASW Statistic 18 (IBM Corp., Armonk, NY, USA).

## 3. Results

More than half (62%) of the referees studied held an R-A license, but the percentage of R-A and R-B licensed referees was comparable (Chi = 1.000; *p* = 0.134). Approximately 68% of the observations analyzed concerned boys matches, with the difference compared to the number of refereed girls matches being insignificant (Chi = 2.250; *p* = 0.134).

First, it was examined whether the assessment of the quality of the referee–players’ interactions is affected by the referee’s license and players’ gender. The results of the tests demonstrated no significant differences in the referees’ ratings between the distinguished groups. Neither the referee license held (Table 4) nor the players’ gender (Table 5) were determinants of the referees’ ratings in the two dimensions studied. These results allowed for the analysis of the ratings of all referees without dividing them into groups according to the independent variables.

The quality of the referee–players’ interactions was measured in two dimensions: building a positive climate and responsiveness to the players’ needs. The first dimension was assessed based on the following indicators: (i) emotional connection; (ii) enthusiasm; (iii) positive comments; (iv) mutual respect. The indicators were measured on a three-point scale indicating poor, average, and good quality of referee–players’ interactions. The results of the observation are presented in Table 6. The majority of referees were rated low on emotional connection (61.9%) and positive comments (76.2%). The quality of other referees’ interactions in these aspects was at an average level. None of the referees studied were rated high on emotional connection, i.e., they were not in physical proximity with the players, did not talk to the players about topics unrelated to the match, and were also not the people the players sought support from. Similarly, no referee received a high score on positive comments, that is, providing verbal and non-verbal support to the players. Referees’ enthusiasm was rated higher than emotional connection and positive comments. Although none of the referees studied was rated high, more than half of the referees received average ratings as they sometimes smiled on the field and reciprocated positive emotions. Others did not do it at all. In terms of building a positive climate, the referees manifested the highest level of quality of interactions in terms of mutual respect. Two-thirds of the observed referees sometimes, but not always, used inclusive and respectful language. Other referees (except for one) displayed this behavior at all times and/or for all players. Individual scores within the indicators of building a positive climate were statistically significant for positive comments (Chi = 4.00; *p* < 0.05) and mutual respect (Chi = 9.875; *p* < 0.05).

The second dimension studied consisted of three indicators: (i) active monitoring of players’ emotional, cognitive, social, and health needs, (ii) responding to the players’ needs, and (iii) solving problems. The majority of the referees received a good rating for indicators 2 and 3. This means that more than half of the referees provided adequate support to the players so that observed problems did not escalate and were not postponed. These referees were always responsive to the players’ needs and efficient at solving problems, so the players felt comfortable and could always count on their support. Other referees either did not always do it or did not do it at all and the problems continued over time with the player’s needs not being met. Nevertheless, in the case of the indicator ‘active monitoring of players’ needs’, which is the most important from the pedagogical point of view, the referees rated ‘poor’, so did not actively monitor the players. The referee does not move between players to look for signs indicating the need for additional support. They were unresponsive when players were trying to get attention. A third of the respondents did this at times, thus earning an average rating. Differences in referees’ ratings on these indicators were significant for the active monitoring of players’ emotional, cognitive, social, and health needs (Chi = 7.625; *p* < 0.05).

Based on the scores for each indicator, ratings for two dimensions were determined. The method of determining these ratings is presented in Table 7. The quality of the referee–players’ interactions was measured on a 7-point scale (1–2 points meant poor; 3–5 points, average; and 6–7 points, good quality of interactions with the players). The observations showed that the average assessment of building a positive climate by referees during a sporting event was ‘poor’ (2.81 pts). The referees were rated significantly higher on the second dimension of responsiveness to the players’ needs (Z = −2.460; *p* = 0.014), although the mean score of 3.81 points indicates an average level of quality of interactions with the players. The result of the Cronbach’s alpha reliability analysis was 0.771.

## 4. Discussion

It was assumed in the research that building a positive climate by the referee and responding appropriately to the needs of young players are important dimensions of the educational function performed by the referee [13]. The main aim of the study was to evaluate the quality of referee–players’ interactions in both the above-mentioned dimensions. Another aim of the study was to examine whether the referee’s qualifications and players’ gender affect the quality of their interactions with the players. The results indicated that neither the referee’s license nor the players’ gender were determinants of the referees’ ratings in any of the dimensions studied. 

Many studies have shown that as experience increases, referee competence improves [43,44,45]. The lack of differences in the ratings for the dimensions studied depending on the license held by the referee may be due to the contents of the referee training, which do not include problems related to pedagogy and developmental psychology of children and youth. The lack of effect of experience on higher ratings of the quality of referee–players’ interactions in terms of building a positive climate and responsiveness to players’ needs has also been observed in studies of soccer, handball, and rugby referees [18,38,39].

Looking at the content of training in Poland, one can see that it is similar across different team sports. This is due to international regulations; currently, most guidelines are created at the level of the relevant international sports federations whereas national federations adapt their guidelines accordingly. To put it in a nutshell, the topics of referee training concern: (1) the rules of the game and their interpretation; (2) physical preparation (training, warm-up, development of general fitness); (3) moving on the field and the techniques of refereeing. Furthermore, courses are increasingly supplemented by meetings with a psychologist who teaches referees how to deal with stress, practice assertiveness, and strengthen mental toughness and self-control. Sometimes, at higher levels, the training topics include body language, managing behavior, building relationships with players and coaches, presenting the coach’s perspective and players’ expectations of referees [46,47,48]. Child protection issues in sport are also discussed during the training of football referees in England. Referees are taught to build a safe, welcoming, and inclusive educational environment [49]. It is found that they play a particular role in this process. In Poland, the training of floorball referees does not include such contents at any stage of their career development.

The lack of differences in the ratings of referees between the groups distinguished by their license may also be due to the fact that the young referees are still full of enthusiasm and enthusiastic about his or her duties, which is positively perceived by the young players. Experienced referees who officiate matches at the highest level can transfer the habits developed and established there to children and youth sport. In adult sports, referees are not expected to build a positive climate and respond to the player’s needs nor are they expected to do so to a lesser degree. Another explanation for the lack of differences in these assessments may simply be an innate teaching talent.

It is commonly believed that the referees perform control, organization, supervision, and order functions [50]. It is not assumed that the referee also has additional goals related to sports education, although the rules of the game themselves have educational values: they usually prohibit unsportsmanlike behavior and instill respect for the opponent. Enforcing them by a referee is already considered an educational activity [13]. However, being aware of the fact that referees are an important element of sports education, they should be required to use a wider range of educational influence, not directly contained in the rules of the game.

A sports field will be more conducive to the multifaceted development of children and youth if there is a caring climate created by all adults [10,14,26,28,31,39]. The results of the study show that referees contributed insignificantly to building such a climate during floorball matches. The quality of the referee–players’ interactions was rated “poor” in this aspect (2.81/7.00 pts.). This means that the referees showed no interest in the players, there was no relationship between each other, no shared joyful moments were observed, and they tended to be at a considerable distance from each other. The referees did not engage in social conversations and clearly lacked enthusiasm and enjoyment related to their work and interactions with players. They rarely gave positive comments to the players and did not display respectful attitudes towards them (calm voice, use of words like “please”, “thank you”, etc.). Comparing these results to findings of studies conducted using the same tool in other sports [13,18,39], it can be concluded that they are similar to those obtained when observing handball referees, whose quality of interactions with players was also rated “poor” on this dimension. Furthermore, in a study of football and rugby, referees were rated at an average level in the dimension of building a positive climate. These differences are probably due to the nature of the sport discussed. With its dynamics, floorball resembles handball more than football or rugby. Football and rugby are slower games, with longer actions, a slower pace, and greater distances between players. In contrast, in handball and floorball, the transition time from defensive to offensive actions is shorter. The game takes place in a relatively small area and with closer distances between players. The referee’s decisions must be made faster, as the stoppage of play can be maximally shortened by the team awarded possession of the ball. The ratings obtained by the referees should not be interpreted as a low level of their competence. They perform their work in accordance with the requirements of the individual organizations managing training and evaluation of referees. The results of the present study indicate areas in which referees can improve. This is because they refer to the postulated referee-educator model proposed by researchers dealing with these problems [13].

Andersson [14] emphasized that referees should equally focus on competition and the spirit of the game in order to support players’ positive experiences of participating in sport. The approach focused on winning at all costs should be replaced in youth sport by the postulate of the joy of the game, the best possible performance of the young player, and their satisfaction with the relationships with those participating and involved in the competitions. A study by Fry and Gano-Overway [28] demonstrated that players who experienced care and support in the sporting environment were willing to reciprocate such behavior towards their teammates, coaches, and referees, derived more enjoyment from sport, and were more committed to the task. Pennington [31] called this building a task-oriented atmosphere.

Researchers emphasize that the building of a positive climate by adults may contribute to the less frequent withdrawal of children from sport [10,11,21,51,52,53]. The role of the referee in creating good relationships with the players during the match is increasingly emphasized. His or her knowledge and skills in this area are crucial for the quality of communication, leadership, and game management [20,44,54,55].

Emotional support of young players also requires the ability to observe and diagnose their emotional states [45]. According to the referees, a good referee is one who can interpret the emotions and behavior of the players, that is, monitor the situation and read the game [54]. In the present research, the responsiveness of floorball referees to the players’ needs was rated as “average” (3.81/7 pts.). This result implies that the referees did not always monitor the players for the need for additional support, especially with regard to their health. They usually tried to meet the perceived players’ needs but did not check the effects of the help given afterwards. The ratings obtained for floorball referees on the responsiveness dimension are similar to those of handball referees [18]. Football and rugby referees were rated higher [38,39] than floorball referees on this dimension. The above results may be due to the nature of the sport being refereed (similar to building a positive climate). Nevertheless, the space for referee improvement was also identified. Situation monitoring, meaning the ability to read players’ intentions and recognize their emotional state, is one of the key competencies of a good referee [43,54,56,57]. In children’s sport, this is particularly important because of the educational goals.

Research on referees in various sports in Poland allows for the creation of an increasingly clear picture of the quality of their educational function. The results of the observations indicate how the contents of referee training can be modified in order for referees to become partners with parents and coaches in creating a positive sporting experience for young players. The ratings obtained by floorball referees represent a diagnosis of the state of their knowledge and pedagogical/psychological skills. The picture of these competencies will be more complete when it is supplemented by examinations of referees in other sports where they interact with athletes in numerous ways (interactor referees). It is still worthwhile to carry out observations of referees, e.g., in combat sports, and to make a more detailed description of their educational function taking into account the specificity of various sports. The study on floorball referees should also be continued due to the small size of the study group. The research discussed in this paper has some limitations related to the direct observation method used. The presence of the observer during the matches may have influenced the referees’ behavior (the Hawthorne effect), but even if the referees tried harder than usual during the observed matches, the ratings are still valuable as they indicate the maximum of their abilities.

## 5. Conclusions

The climate of a sporting event is critical to the players’ experiences, and the behavior of the fans. The referee, who is the only adult on the field during children’s matches, can play an important role here. Building a positive climate and responding to players’ needs are considered important dimensions of the educational function of a referee. The referee’s enthusiastic attitude towards players, use of verbal and non-verbal positive reinforcement, emotional closeness to the players, responding to their health, cognitive, and social needs, respect for the players, coaches, and spectators are crucial for the referee to utilize the educational potential of sport. The results of the present study lead to the following conclusions: (i) the contents of training for floorball referees should include problems of pedagogy and developmental psychology; (ii) referees should be equipped with appropriate competencies for building a positive climate during matches and monitoring the players’ needs; (iii) referees appointed to officiate children and youth games should be characterized by appropriate predispositions.

## Figures and Tables

**Table 1:** Descriptions of indicators of building a positive climate [18].

Indicators	Quality of Referee–Players’ Interaction		
	Poor	Average	Good
Emotional connection - physical proximity - social conversation - the players seek support from the referee	Clear physical and emotional distance between the referee and players is observed. In addition to the messages related to the game, the referee does not talk to the players.	It can be observed that the referee and the players show mutual interest, but this only applies to one team or individual players. A physical and emotional distance between the referee and the players is sometimes observed.	The referee shows great interest in all players. Physical contact and emotional closeness are observed. Their relationship is warm and supportive. The referee sometimes talks to the players about problems unrelated to the game.
Enthusiasm - smiling - engagement - positive affective reaction	The referee does not show an enthusiastic attitude towards the players and his or her duties. They do not smile at all and do not reciprocate the positive emotions of the players.	The referee is enthusiastic and smiles, but there are moments when he or she does not do this or not to all players. The referee sometimes reciprocates the positive emotions of the players.	The referee shows an enthusiastic attitude and often smiles. He or she always reciprocates the positive emotions of the players.
Positive comments (verbal and non-verbal)	The referee does not give positive comments to the players at all.	The referee sometimes gives positive comments to the players or does it often, but they are apparently insincere. The referee gives positive comments to only one team or selected players.	The referee often gives positive comments to all players and they are apparently sincere and unforced.
Mutual respect - respectful and inclusive language - using players first names - calm voice - listening to players	The referee and players rarely, if ever, demonstrate respect for one another. Competitors do not recognize the authority of the referee, often questioning his or her decision.	The referee and players sometimes demonstrate respect for one another; however, these interactions are not consistently observed across time or players and it happens that the players question the referee’s authority.	The referee and players consistently demonstrate respect for one another. The referee has the authority and his/her decisions are not called into question.

**Table 2:** Descriptions of indicators of responsiveness to the players’ needs [18].

Indicators	Quality of Referee–Players’ Interaction		
	Poor	Average	Good
Active monitoring of players’ emotional, cognitive, social and health needs	The referee does not monitor the players to meet their needs and does not know when the players need additional support or help.	The referee sometimes monitors the players to meet their needs and notices when they need extra support or help, but there are moments when this does not happen.	The referee constantly monitors the players to meet their needs and always notices when they need additional support or help.
Responding to the players’ needs - fast meeting of the players’ needs	The referee does not respond to or neglects the players’ needs.	The referee sometimes responds to the players’ needs, or this reaction does not apply to everyone.	The referee always responds to the social, emotional, and health needs of the players.
Solving problems	The referee cannot solve a problem that goes on and on.	The referee attempts to solve the problem, but he or she does not always do it effectively.	The referee manages to solve the problems that arise and they do not last long.

**Table 3:** The way to determine the assessment of each dimension [18].

Three Indicators	Four Indicators	Score	
P, P, P	P, P, P, P	1	POOR
P, P, A	P, P, P, A	2	
P, A, A	P, P, A, A P, A, A, A	3	AVERAGE
A, A, A P, A, G	P, A, A, G A, A, A, A	4	
A, A, G	A, A, A, G A, A, G, G	5	
A, G, G	A, G, G, G	6	GOOD
G, G, G	G, G, G, G	7	

Abbreviations: P = poor; A = average; G = good.

**Table 4:** Differences in the assessment of the quality of referee–players’ interactions in two dimensions depending on the referee license held (*n* = 21).

	Referee License					
Dimensions	R-B License [40] Referees Who Completed the Refereeing Course of the Polish Floorball Federation and Practical Experience of Performing the Role of a Referee, Confirmed by the Observer’s Opinion. (*n* = 8)		R-A License [40] Referees with Refereeing Experience Who Had Officiated Matches for at Least Several Seasons Across all Age Categories. (*n* = 13)		Mann–Whitney U Test	
	Median	$\bar{x}$ Rang.	Median	$\bar{x}$ Rang.	Z	*p*
Building a positive climate	2.50	8.58	2.50	8.48	−0.057	0.958
Responsiveness to the players’ needs	4.00	8.17	4.40	8.70	−0.222	0.825

**Table 5:** Differences in the assessment of the quality of referee–players’ interactions in two dimensions depending on the refereed girls’ or boys’ matches (*n* = 21).

Dimensions	Girls Matches (*n* = 7)		Boys Matches (*n* = 14)		Mann–Whitney U Test	
	Median	$\bar{x}$ Rang.	Median	$\bar{x}$ Rang.	Z	*p*
Building a positive climate	2.00	11.60	4.00	7.09	−0.1.851	0.090
Responsiveness to the players’ needs	4.00	8.80	4.00	8.36	−0.174	0.913

**Table 6:** Assessment the quality of referee–players’ interactions in the dimension of *building a positive climate* and *responsiveness to players’ needs* during children’s floorball competitions (*n* = 21).

			Quality of Referee–Players’ Interactions			Chi-Squared Test *p*
Dimension		Indicators	Poor	Average	Good	
Building a positive climate		Emotional connection (physical proximity, social conversation, the players seek support from the referee)	61.9%	38.1%	0.0%	0.317
		Enthusiasm (smiling, engagement, positive affective reaction)	42.9%	57.1%	0.0%	0.617
		Positive comments (verbal and non-verbal)	76.2%	23.8%	0.0%	0.046 *
		Mutual respect (respectful and inclusive language, using players first names, calm voice listening to players)	4.8%	66.7%	28.6%	0.007 *
Responsiveness to the players’ needs	Active monitoring of players’ emotional, cognitive, social, and health needs		61.9%	33.3%	4.8%	0.022 *
	Responding to the players’ needs (fast meeting of the players’ needs)		19.0%	23.8%	57.1%	0.144
	Solving problems		23.8%	23.8%	52.4%	0.368

* *p* < 0.05.

**Table 7:** Descriptive statistics and normality assessment for the ratings of quality of referee–players’ interactions in *building* a *positive climate* and *responsiveness*.

Dimension	Mean (SD)	Median	Shapiro–Wilk *p*
Building a positive climate	2.81 (1.2)	2.5	0.031
Responsiveness to the players’ needs	3.81 (1.6) *	4.0	0.567

* significantly (*p* < 0.05) higher than in the building a positive climate (Wilcoxon’s signed ranks test).

## References

[1] Hellison D. (2011). Teaching Personal and Social Responsibility through Physical Activity.

[2] Cronin L.D., Allen B.A. (2015). Developmental experiences and well-being in sport: The importance of the coaching climate. Sport Psychol..

[3] Light R.L., Harvey S. (2015). Positive pedagogy for sport coaching. Sport Educ. Soc..

[4] Thompson J. (2013). The Double-Goal Coach: Positive Coaching Tools for Honoring the Game and Developing Winners in Sports and Life.

[5] Camiré M., Forneris T., Trudel P., Bernard D. (2011). Strategies for helping coaches facilitate positive youth development through sport. J. Sport Psychol. Action.

[6] Horn T.S., Horn T.S. (2008). Coaching effectiveness in the sport domain. Advances in Sport Psychology.

[7] Romani Q.A. (2019). Parental behaviour and children’s sports participation: Evidence from a Danish longitudinal school study. Sport Educ. Soc..

[8] Strandbu Å., Stefensen K., Smette I., Sandvik M.R. (2019). Young people’s experiences of parental involvement in youth sport. Sport Educ. Soc..

[9] Anderson J.C., Jeanne B.F., Elliott R., Smith P.H. (2003). Parental support and pressure and children’s extracurricular activities: Relationships with amount of involvement and affective experience of participation. J. Appl. Dev. Psychol..

[10] Cumming S.P., Smoll F.L., Smith R.E., Grossbard J.R. (2007). Is winning everything? The relative contributions of motivational climate and won-lost percentage in youth sport. J. Appl. Sport Psychol..

[11] Pelletier L.G., Fortier M.S., Vallerand R.J., Briere N.M. (2002). Associations among perceived autonomy support, forms of self-regulation, and persistence: A prospective study. Motiv. Emot..

[12] Barnett N.P., Smoll F.L., Smith R.E. (1992). Effects of enhancing coach-athlete relationships on youth sport attrition. Sport Psychol..

[13] Firek W., Płoszaj K. (2021). Edukacyjna Funkcja Sędziego w Sporcie Dzieci i Młodzieży [Educational Function of Referee in Children and Youth Sport].

[14] Andersson E. (2019). A referee perspective on the educational practice of competitive youth games: Exploring the pedagogical function of parents, coaches and referees in grassroots soccer. Phys. Educ. Sport Pedagog..

[15] Isidori E., Müller A., Kaya S. (2012). The Referee as Educator: Hermeneutical and Pedagogical Perspectives. Phys. Cult. Sport Stud. Res..

[16] MacMahon C., Mascarenhas D., Plessner H., Pizzera A., Oudejans R.R.D., Raab M. (2015). Sports Officials and Officiating: Science and Practice.

[17] Plessner H., MacMahon C., Farrow D., Baker J., MacMahon C. (2013). The sports official in research and practice. Developing Sport Expertise: Researchers and Coaches Put Theory into Practice.

[18] Płoszaj K., Firek W., Czechowski M. (2020). The Referee as an Educator: Assessment of the Quality of Referee–Players Interactions in Competitive Youth Handball. Int. J. Environ. Res. Public Health.

[19] Wheeler S., Green K. (2014). Parenting in relation to children’s sports participation: Generational changes and potential implications. Leis. Stud..

[20] Arthur-Banning S.G., Paisley K., Wells M.S. (2007). Promoting Sportsmanship in Youth Basketball Players: The Effect of Referees’ Prosocial Behavior Techniques. J. Park Recreat. Adm..

[21] Crane J., Temple V. (2015). A systematic review of dropout from organized sport among children and youth. Eur. Phys. Educ. Rev..

[22] Montesano P., Tafuri D., Mazzeo F. (2017). The drop-outs in young players. J. Phys. Educ. Sport.

[23] Elliott S.K., Drummond J.N. (2017). Parents in youth sport: What happens after the game?. Sport Educ. Soc..

[24] Smoll F.L., Smith R.E., Cumming S.P. (2007). Effects of a motivational climate intervention for coaches on changes in young athlete’s achievement goal orientations. J. Clin. Sport Psychol..

[25] Cox A., Williams L. (2008). The roles of perceived teacher support, motivational climate, and psychological need satisfaction in students’ physical education motivation. J. Sport Exerc. Psychol..

[26] Olympiou A., Jowett S., Duda J.L. (2008). The psychological interface between the coach-created motivational climate and the coach-athlete relationship in team sports. Sport Psychol..

[27] Duda J.L., Ntoumanis N., Mahoney J.L., Larson R.W., Eccles J.S. (2005). After-school sport for children: Implications of a task-involving motivational climate. Organized Activities Ascontexts of Development.

[28] Fry M.D., Gano-Overway L.A. (2010). Exploring the contribution of the caring climate to the youth sport experience. J. Appl. Sport Psychol..

[29] Newton M., Fry M., Watson D., Gano-Overway L., Kim M., Magyar M., Guivernau M. (2007). Psychometric properties of the caring climate scale in a physical activity setting. Rev. Psicol. Deporte.

[30] Gano-Overway L.A., Newton M., Magyar T.M., Fry M., Kim M., Guivernau M. (2009). Influence of caring youth sport contexts on efficacy-related beliefs and social behaviors. Dev. Psychol..

[31] Pennington C.G., Pennington C.G. (2019). Observing Positive Sporting Behaviors during Golf Competition Using the THOMAS Instrument. Moral Development and Behavior: New Research.

[32] Lavay B.W., French R., Henderson H.L. (2016). Positive Behavior Management in Physical Activity Settings.

[33] Treasure D., Roberts G.C. (2001). Enhancing young people’s motivation in youth sport: An achievement goal approach. Advances in Motivation in Sport and Exercise.

[34] Allender S., Cowburn G., Foster C. (2006). Understanding participation in sport and physical activity among children and adults: A review of qualitative studies. Health Educ. Res..

[35] Adachi P.J.C., Willoughby T. (2014). It’s not how much you play, but how much you enjoy the game: The longitudinal associations between adolescents’ self-esteem and the frequency versus enjoyment of involvement in sports. J. Youth Adolesc..

[36] Visek A., Achrati S.M., Manning H., McDonnell K., Harris B.S., DiPietro L. (2015). The fun integration theory: Towards sustaining children and adolescents sport participation. J. Phys. Act. Health.

[37] Babbie E. (2001). The Practice of Social Research.

[38] Firek W., Płoszaj K., Czechowski M. (2020). Pedagogical Function of Referees in Youth Sport: Assessment of the Quality of Referee-Player Interactions in Youth Soccer. Int. J. Environ. Res. Public Health.

[39] Płoszaj K., Firek W., Ciszewska-Hołda P. (2021). Assessment of Referees in Terms of Building a Positive Climate and Responsiveness to the Health, Emotional, and Social Needs of Rugby Players in Competitive Children Sport. Int. J. Environ. Res. Public Health.

[40] PFF (2020). Regulamin Kategorii Sędziowskich. http://www.polskiunihokej.pl/pzunihokeja/wydzia%C5%82-s%C4%99dziowski/regulaminy-ws#.

[41] Bryman A. (2012). Social Research Method.

[42] Kothari C.R. (2004). Research Methodology. Methods and Techniques.

[43] MacMahon C., Helsen W.F., Starkes J.L., Weston M. (2007). Decisionmaking skills and deliberate practice in elite association football referees. J. Sports Sci..

[44] Simmons P., Tebbutt J. (2008). Fair call-player perceptions of justice in football referee communication. ANZCA2007 Communications, Civics, Industry.

[45] Dosseville F., Laborde S., Garncerzyk C., Mohiyeddini C. (2013). Current research in sports officiating and decision-making. Contemporary Topics and Trends in the Psychology of Sports.

[46] EHF Guidelines for Referee Education. European Handball Federation EHF Competence Academy & Network Recruitment, Promotion and Advancement on EHF and National Level, Vienna, 2011. http://ebook.eurohandball.com/2011EHFGuidelinesforRefereeEducation/html/3.html.

[47] Holdhaus H. (2011). Shuttle Run Test and Training Advice, Guidelines for Referee Education 2011.

[48] Acsinte A. Referees–Coaches. 12th EHF Referee Candidates Course 13–16 June 2013 Drammen. 2013. http://activities.eurohandball.com/ehfcan/16054.

[49] FA FA Basic Referee Training Course. 2020. http://www.thefa.com/get-involved/referee/general-information/the-fa-basic-referee-training-course.

[50] Żyto S. (2010). Polifunkcyjny wymiar aktywności sędziów sportowych [The polyfunctional dimension of the activity of sport referees]. Sport Wyczyn..

[51] Sarrizin P., Vallerand R.J., Guillet E., Pelletier L.G., Cury F. (2002). Motivation and dropout in female handballers: A 21-month prospective study. Eur. J. Soc. Psychol..

[52] Wagnsson S., Gustafsson H., Libäck J., Podlog L.W. (2021). Lessons Learned from a Multi-Level Intervention Program to Reduce Swedish Female Floorballers’ Dropout Rate. J. Sport Psychol. Action.

[53] Witt P.A., Dangi T.B. (2018). Why Children/Youth Drop Out of Sports. J. Park Recreat. Adm..

[54] Cunningham I., Simmons P., Mascarenhas D.R.D., Redhead S. (2014). Skilled Interaction: Concepts of communication and player management in the development of sports officials. Int. J. Sport Commun..

[55] Cunningham I., Simmons P., Mascarenhas D., Redhead S. (2015). Exploring player communication in interactions with sport officials. Mov. Sports Sci. Sci. Mot..

[56] Morris P.H., Lewis D. (2010). Tackling diving: The perception of deceptive intentions in Association Football (soccer). J. Nonverbal Behav..

[57] Płoszaj K., Firek W. (2021). Self-assessment of football referees on their educational influence on children aged 9–12 during a sports competition. Phys. Cult. Sport Stud. Res..

